# *Lin28b-let-7* Modulates mRNA Expression of *GnRH1* Through Multiple Signaling Pathways Related to Glycolysis in GT1-7 Cells

**DOI:** 10.3390/ani15020120

**Published:** 2025-01-07

**Authors:** Yujing Xie, Xin Li, Meng Wang, Mingxing Chu, Guiling Cao

**Affiliations:** 1 School of Agriculture and Biology, Liaocheng University, Liaocheng 252059, China13210343339@163.com (X.L.);; 2 State Key Laboratory of Animal Biotech Breeding, Institute of Animal Science, Chinese Academy of Agricultural Sciences, Beijing 100193, China

**Keywords:** *Lin28b-let-7*, GnRH, glycolysis, MAPK, mTOR

## Abstract

*Lin28b* and *let-7* miRNA regulate mammalian pubertal initiation and Gonadotropin-releasing hormone (GnRH) production. However, it remains unclear which signaling pathways *Lin28b* regulates to modulate GnRH production. In this study, the mRNA expression levels of *Lin28b* and *let-7* in the pubertal and juvenile goat hypothalamus and pituitary gland were detected, and *Lin28b* expression in the pubertal hypothalamus decreased significantly compared with that in juvenile tissues. It was predicted that *Lin28b* might inhibit *GnRH1* expression, which was verified in the GnRH-producing cell model GT1-7 cells. *Lin28b* inhibited *GnRH1* expression and promoted Kiss1/Gpr54 signaling. The pyruvate content and the expression of *Hif1a* and *Hk2*, which were related to glycolysis, were also promoted by *Lin28b* overexpression. Additionally, 77 differentially expressed miRNAs (DEMIs) in *Lin28b*-overexpressed GT1-7 cells were identified. Bioinformatics analysis and mRNA expression of the target genes of DEMIs revealed that the MAPK and PI3K-AKT-mTOR signaling pathways were key pathways that involved the regulatory effect of *Lin28b* on GnRH. In GT1-7 cells, *GnRH1* expression was suppressed by blocking mTOR signaling with rapamycin, which was rescued by *Lin28b* overexpression. These results indicate that *Lin28b-let-7* regulates *GnRH1* expression through several pathways, including the Kiss1/Gpr54, MAPK, and mTOR signaling pathways, which are all related to glucose metabolism and provide new insights into the molecular mechanism of the regulatory role of *Lin28b* on GnRH production.

## Simple Summary

Gonadotropin-releasing hormone (GnRH) production is the key regulator for reproduction, containing puberty initiation, which is regulated by *Lin28b* and *let-7* miRNA. However, the regulatory mechanism of *Lin28b* on GnRH remains unclear. In this study, a decreased *Lin28b* expression was found in the hypothalamus of pubertal goats. In the GnRH-producing cell model GT1-7 cells, *Lin28b* inhibited *GnRH1* mRNA expression and promoted glycolysis and Kiss1/Gpr54 signaling. Seventy-seven differentially expressed miRNAs containing *let-7* were selected in GT1-7 cells with *Lin28b* overexpression through small RNA-seq. Target genes analysis and RT-qPCR revealed the importance of the MAPK and PI3K-AKT-mTOR signaling pathways. And in GT1-7 cells, *Lin28b* overexpression rescued the *GnRH1* expression suppressed by blocking mTOR signaling with rapamycin. In summary, *Lin28b* modulates GnRH expression through several signaling pathways related to glucose metabolism and at least partially through *let-7*. This study provides new insights into the mechanism of GnRH production.

## 1. Introduction

The pulsatile release of GnRH is the key driver of mammalian pubertal onset [1]. GnRH stimulates the secretion of follicle-stimulating hormone (FSH) and luteinizing hormone (LH) from the anterior pituitary gland. FSH and LH initiate gametogenesis and steroid hormone synthesis. At the onset of puberty, the pulsatile release of GnRH is manipulated by GnRH neurons distributed in the arcuate nucleus and preoptic area of the hypothalamus and controlled by a complex network, which is composed of various excitatory and inhibitory neurotransmitters and neuropeptides [2]. Pubertal GnRH neurons are highly sensitive to energy levels and metabolic status and are modulated by hormones related to energy metabolism, such as leptin and insulin [3,4].

*Lin28a* and *Lin28b*, homologs of the *Caenorhabditis elegans* heterochronic gene *Lin28*, encode RNA-binding proteins, which can disturb the maturation process of *let-7* microRNAs (miRNAs) or modulate mRNA translation [5], thereby regulating the self-renewal of mammalian embryonic stem cells, organismal growth and metabolism, somatic reprogramming and cancer. Several studies reported that *Lin28a/b* regulated the pubertal age and pubertal body weight of mice in a sex-specific pattern [6,7]. The expression of *c-Myc/Lin28a/let-7* changed in the hypothalamus of rats with pubertal delay caused by malnutrition [8]. The central administration of the Lin28b protein stimulated the synthesis of dynorphin, a known inhibitor of GnRH neurons, but did not alter the expression of kisspeptins, which were encoded by the *Kiss1* gene and bound to their receptor Gpr54 in GnRH neurons to promote GnRH secretion [9,10]. *Lin28a* overexpression in GT1-7 cells reduced the GnRH concentration and *Kiss1* mRNA expression [11]. In mammals, *Lin28a* and *Lin28b* were expressed in a variety of tissues during early embryonic development but were restricted to some kinds of adult tissues, such as the hypothalamus, pituitary, ovary, and testis, which are related to reproduction [6,12,13,14]. Additionally, in the pubertal hypothalamic tissues of mice [14], rats, and monkeys [8], the expression of *Lin28a* was significantly lower than that in juvenile tissues. *Lin28b* has a similar expression pattern in the hypothalami of rats and monkeys [8]. Taken together, these findings reveal that *Lin28a* and *Lin28b* exert a prepubertal inhibitory effect, but the molecular mechanism involved remains unclear. Notably, several independent genome-wide association studies reported that several mutations close to or within *Lin28b* were significantly associated with the age at menarche in girls [15,16,17,18,19,20].

*Lin28a/b* regulate glucose metabolism through *let-7* miRNA, insulin-phosphatidylinositol 3′-kinase (PI3K), mammalian target of rapamycin (mTOR) signaling pathway, and mitogen-activated protein kinases (MAPK) signaling pathway [21,22,23,24]. PI3K and mTOR signaling pathways are the major molecular pathways associated with metabolic regulation, leptin signaling, insulin signaling, glucose homeostasis, and neuroendocrine function. The mice with deletion of PI3K p110α catalytic subunit in leptin receptor cells showed delayed puberty. Blockade of central mTOR signaling in rats caused delayed onset of puberty [25]. Several key genes in the PI3K/AKT/mTOR and the adenosine monophosphate (AMP)-activated protein kinase (AMPK) signaling pathways, which are significant signaling pathways linking energy and reproduction, are the target genes of *Lin28a/b* and/or *let-7* miRNAs [22,26,27]. Therefore, we speculate that *Lin28a/b* participates in the onset of puberty or GnRH secretion by regulating glucose metabolism.

Goats are a good model for studying sexual maturation. The degree of sexual development attained by the goat kid in slightly greater than 6 months requires 13–15 years in the human child. Thus, the compressed developmental period of the goat kid facilitates studies of the sexual maturation of large, long-lived mammalian females. In our previous study, some mutations in *Lin28b* showed different allele gene frequencies in sexual precocious goat breeds and late-maturing breeds. To gain insight into the physiological role of *Lin28b* in GnRH expression, the *Lin28b* and *let-7* expression in the hypothalamus between juvenile and pubertal goats were compared. Next, we altered the expression of *Lin28b* in GT1-7 cells to analyze the changes in *GnRH1*, *Kiss1*, and *Gpr54* expression to verify whether *Lin28b* inhibits *GnRH1* expression via Kiss1/Gpr54 signaling. Moreover, the content of glycolysis products and the expression of related genes were detected to determine whether glucose metabolism participates in the regulatory effect of *Lin28b* on *GnRH1* expression. The miRNA expression patterns were subsequently analyzed via small RNA sequencing, and the mRNA abundance of candidate genes was analyzed via quantitative PCR to screen key signaling pathways. Moreover, the regulatory effect of *Lin28b* on the effect of mTOR signaling on GnRH production was also investigated. The schematic diagram is shown in Supplementary Figure S1. This study provides new insights into the regulatory mechanism of energy on pubertal onset or GnRH secretion.

## 2. Methods and Materials

### 2.1. Ethics Approval

All procedures involving animals were approved by the Animal Care and Use Committee of Liaocheng University (the approval number: 2023032801, 28 March 2023) (Liaocheng, Shandong Province, China). And all efforts were made to minimize animal suffering.

### 2.2. Experimental Animals and Tissues Collection

Jining Grey (JG) goats were selected as the experimental animals. JG goat is a unique local breed in China that displays significant characteristics of sexual precocity; pubertal initiation occurs at 2–3 months old. Animal experiment was carried out from May to July in Heze city (35° N, 115° E), Shandong Province, China. A total of 30 clinically normal female 1-month-old Jining Grey (JG) goats with similar body weights were selected and housed in open sheepfolds and fed ad libitum. At 6:00–7:00 a.m. and 6:00–7:00 p.m., the behavior, sexual receptivity, and interest in vasectomized teaser bucks were noted daily from 1.5 months old. Furthermore, external genitalia and secretion were checked to determine the estrus. The female goat kids showing estrus behaviors, such as appearing excited, always looking around, going after the buck, and accepting the mounting of the buck first time, were considered to reach puberty. And tissue samples were collected when the female kids were during the first estrus cycle of puberty.

**Figure 1:**
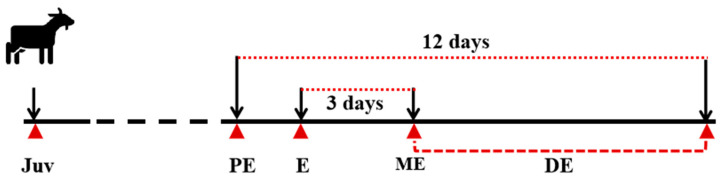
The diagram of sample collection. Red triangles represent the day that goats were sacrificed. Juv, juvenile group, 1-month-old goats. PE, goats that were in proestrus. E, goats that were in estrus. ME, metestrus goats. DE, goats that were in diestrus.

As shown in Figure 1, the goats were scheduled for slaughter on days of proestrus (the first day that female goats display obvious estrus signals, such as appearing excited, looking around, and small amounts of thin mucus on the vulva. An amount of n = 3, PE, BW, 7.83 ± 0.48 kg), estrus (the first day that female goat kids accept mounting of the teaser bucks, a large amount of transparent and thin mucus on vulva. n = 3, E, BW, 8.13 ± 0.56 kg), metestrus (the third day from E day, refusing mounting of the teaser bucks and a thin and thick mucus on vulva. n = 3, ME, BW, 8.06 ± 0.33 kg), and diestrus (the twelfth day from PE day, n = 3, DE, BW, 8.64 ± 0.64 kg). In addition, three 1-month-old female JG goats, weighting 5.06 ± 0.44 kg, were determined without estrus and named as Juvenile (JUV) group. Goats of each group were sacrificed at 5:00–6:00 p.m. after anesthesia [28,29]. Before slaughter, goats were fasted for 10–12 h or 24 h with free access to water. Goats were administered with xylazine hydrochloride (500 μg/kg body weight) intravenously (IV), and after 10 min, the goats were immediately euthanized with IV sodium pentobarbital (100 mg kg^−1^). The hypothalamus and pituitary tissues were collected and preserved in RNAlater RNA Stabilization reagent (Qiagen, Hilden, Germany, 76106) and kept at −20 °C for RNA isolation.

### 2.3. Cell Culture

GT1-7 cells, kindly provided by Prof. T Feng (Beijing Academy of Agriculture and Forestry Sciences, Beijing, China), under the authorization of Prof. Pamela Mellon from the University of California, San Diego, CA, USA were grown in Dulbeco’s Modified Eagle’s Medium (DMEM) (Gibco, Waltham, MA, USA, C11995500) with 10% fetal bovine serum (Gibco, 10099-141), penicillin (100 U/mL), and streptomycin (100 U/mL) and maintained at 37 °C with 5% CO_2_. For serum starvation, the cells were plated at 60% confluency, grown overnight, and changed to serum-free culture media, and the incubation was continued for 6–48 h, depending on the experimental design.

### 2.4. Cell transfection and Rapamycin Treatment

The overexpression vector pEGFP-gLin28b and the pSilencer-mLin28b interference vector were constructed and transfected into GT1-7 cells incubated in the serum-free media, respectively, using Lipofectamine 3000 (Invitrogen, Waltham, MA, USA, L3000-015) according to the manufacturer’s instructions and the empty vector plasmids were also transfected as control. For convenience, the GT1-7 cells transfected with pEGFP-gLin28b and pSilencer-mLin28b were simply named Lin28b+ and Lin28b− cells, respectively. Then, 48 h after transfection, the cells culture supernatant and cells were collected to detect GnRH concentration and pyruvate and ATP content, respectively. Meanwhile, cells were collected to detect the mRNA abundance of *Lin28b*, *GnRH1*, *Kiss1*, *Gpr54*, *Pdk2*, *Hk2*, *Hif1a*, *let-7a/c/e/g*, *Map4k3*, *Map2k4*, *Mapk9*, *JunD*, *Fgf1*, *Erbb2*, *Mapk1*, *Atf4*, *Kcnj3*, *InsR*, *Lepr*, *Ccnd2*, *Pik3r1*, *Akt1*, *mTOR*, and *S6k* by quantitative reverse-transcriptase PCR (RT-qPCR). The cells of Lin28b+ and the control group were also used to perform small RNA sequencing. To investigate the regulatory effect of the mTOR signaling on *GnRH1* expression, mTOR signaling inhibitor ramamycin (MCE, Mooloolaba, Australia, AY-22989) was added to the control GT1-7 cells, and *Lin28b* overexpressed cells with a final concentration 200 μM or 500 μM. The *Lin28b* overexpressed GT1-7 cells treated with 200 μM or 500 μM rapamycin simultaneously were simply named Lin28b+ 200R or Lin28b+ 500R cells. And cells were harvested after 24 h for gene expression detection.

### 2.5. Cells Treatment for GnRH Concentration, Pyruvate and ATP Content Detection

The GnRH concentration, pyruvate, and ATP content were detected using a mouse GnRH ELISA Kit (TSZ, Hong Kong, China, HG32103), pyruvate assay kit (Solarbio, Beijing, China, BC2205) and ATP assay kit (Solarbio, BC0300), respectively. For GnRH concentration, the cell culture supernatant was collected and centrifuged for 20 min at 1000× *g* and assayed immediately or aliquot and stored samples at −80 °C. For pyruvate content detection, the number of cells were harvested and washed with cold PBS. After centrifuging, the cells were collected and added 1mL extracting solution and were shattered using ultrasonication (ice bath, 200 W, 3 s and 10 s intervals, repeated 30 times). Subsequently, the samples were incubated at room temperature for 30 min and centrifuged for 10 min at 8000× *g*. The supernatant was collected to detect the pyruvate content. Similarly, for the ATP assay, the cells resuspended in 1 mL extracting solution were shattered using ultrasonication (ice bath, 200 W, 2 s). Then, samples were centrifuged for 10 min at 4 °C at 10,000× *g* to collect supernatant. Subsequently, 500 μL chloroform was added to the supernatant and fully mixed, centrifuged for 3 min at 4 °C at 10,000× *g*. After that, the supernatant was collected to detect ATP content by ultraviolet spectrophotometry.

### 2.6. RNA Isolation and RT-qPCR

Total RNA was extracted from the hypothalamus and pituitary tissues, and GT1-7 cells using TRIzol and treated with DNase I (RNase free) to remove genomic DNA. For assays of miRNA levels, small RNA was extracted with the RNAiso for small RNA (Takara, San Jose, CA, USA, 9753) according to the manufacturer’s instructions. The procedures for small RNA isolation were similar to the TRIzol method, and the TRIzol was replaced with RNAiso. For the mRNA expression detection, cDNA was generated using the PrimerScript^TM^ RT kit (Taraka, RR037). For miRNA quantification, cDNA was synthesized by using mir-X miRNA First-strand synthesis and TB green RT-qPCR (Clontech, Palo Alto, CA, USA, 638314). QPCR was performed using SYBR Green qPCR Master Mix (Thermo Fisher, Waltham, MA, USA, 4385610) on Applied Biosystems (ABI) CFX96 Touch Real-Time Detection System. The 2^−ΔΔCt^ method was used for data analysis. *Gapdh* and *U6* were used to normalize the target genes and miRNA data, respectively. The primers used for the qPCR assay were provided in Supplementary Table S1.

### 2.7. Library Construction and Small Sequencing

The Small RNA-seq analysis was performed by BGI tech (Shenzhen, China). Briefly, six cDNA libraries were generated from Lin28b+ cells and control cells. Total RNA was isolated using the TRIzol method, and RNA quantification and qualification were tested by Agilent bioanalyzer 2100 before constructing small RNA libraries. The 18–30 nt small RNA was purified with PAGE gel and connected with 3′- and 5′-adapter. The small RNAs ligated with adapters were reverse transcribed into cDNA, which would be denatured into a single strand and cyclized. Single-stranded circle DNA molecules were replicated via rolling cycle amplification to generate DNA nanoballs (DNBs), which contain multiple copies of DNA. Then, sufficient quality DNBs were loaded into patterned nanoarrays using a high-intensity DNA nanochip technique and sequenced through combinatorial Probe-Anchor Synthesis.

### 2.8. Small RNA Annotation and miRNA Identification

From the sequenced, only clean reads were used for downstream analysis, and clean tags were obtained using SOAPnuke (BGI, Shenzhen, China). After filtering, the clean tags were mapped to the reference genome (*Mus musculus*, GCF_000001635.26_GRCm38.p6), miRbase (V22), and other sRNA databases with Bowtie 2 [30]. Particularly, cmsearch was performed for Rfam mapping [31]. Potential novel miRNAs were identified using the miRNA package.

### 2.9. Differential Expression Analysis of miRNA and Target Gene Analysis

The small RNA expression level was calculated by counting absolute numbers of molecules using unique molecular identifiers [32]. Differential expression analysis was performed using DESeq2 [33]. The *Q* values were modified by the *p* values, and *Q* values ≤ 0.05 and |Log_2_FoldChange| ≥ 1 were the default threshold to judge the significance of expression difference. The psRobot [34] and TargetFinder [35] were used to predict the target genes of miRNAs. Target genes were aligned against the Kyoto Encyclopedia of Genes and Genomes (KEGG, Kyoto, Japan, v89.1) and Gene Ontology (GO) (http://www.geneontology.org/, accessed on 5 March 2024) database to annotate gene functions. The *p*-value was corrected using the Bonferroni method. GO, or KEGG terms, fulfilling corrected *p*-value ≤ 0.05, were defined as significantly enriched terms.

### 2.10. Statistical Analysis

The GraphPad Prism (version 9.3.1) software was used for the analysis of RT-qPCR results. Data were shown as mean ± SEM. Unpaired Student’s *t*-tests were used to compare two groups, and one-way ANOVA followed by the pairwise Tukey test was used for multiple comparisons. *p* < 0.05 indicated statistical significance.

## 3. Results

### 3.1. Expression of Lin28b, GnRH1, and let-7b/g in the Hypothalamus and/or Pituitary of Goats During Postnatal Maturation

**Figure 2:**
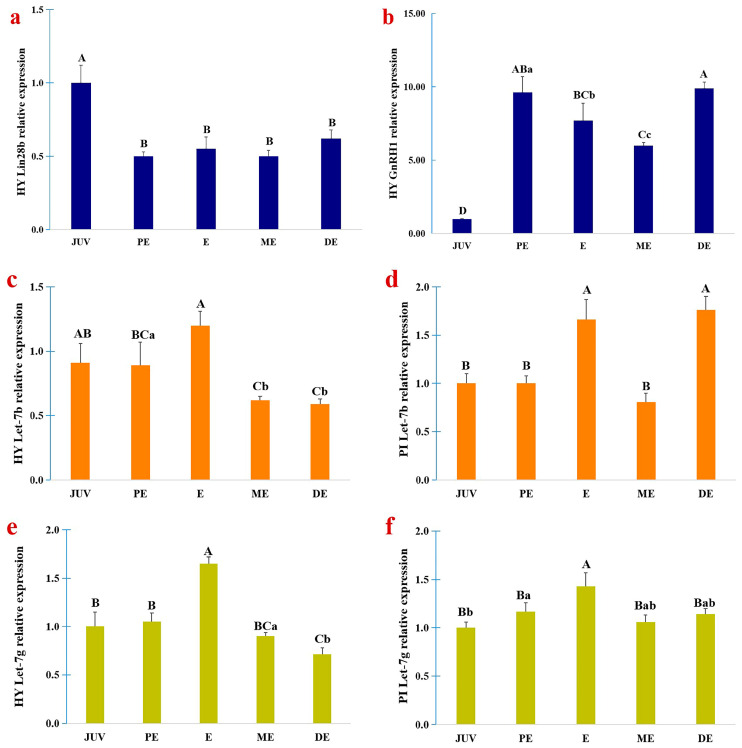
The expression of *Lin28b*, *GnRH1 let-7b*, and *let-7g* miRNA in the hypothalamus and (or) pituitary of goats. (**a**), *Lin28b* and (**b**), *GnRH1* in the hypothalamus. (**c**,**d**), *let-7b* in the hypothalamus and pituitary. (**e**,**f**), *let-7g* in the hypothalamus and pituitary. The different capital letters above the columns chart mean a significant difference with *p* < 0.01, and the different small letter means a significant difference with *p* < 0.05 in multiple comparisons. PE, proestrus. E, estrus. ME, metestrus. DE, diestrus. HY, hypothalamus. PI, pituitary.

The mRNA abundances of *GnRH1*, *Lin28b*, and *let-7b/g* in the hypothalamus and pituitary of juvenile and pubertal goats were detected via RT-qPCR (Figure 2). *Lin28b* expression was significantly downregulated in the pubertal hypothalamus compared with the juvenile hypothalamus (*p* < 0.01) (Figure 2a), which was an opposite expression pattern compared with the expression of *GnRH1* (Figure 2b). The *Lin28b* mRNA abundance in the hypothalamus of proestrus (PE), estrus (E), metestrus (ME), and diestrus (DE) goats showed no significant difference (Figure 2a). However, *GnRH1* expression had obviously changed during the estrus cycle (Figure 2b). *Lin28b* was not expressed or expressed at extremely low levels in the pituitary tissues.

*Let-7b* and *let-7g* in the hypothalamus exhibited similar expression patterns (Figure 2c,e). Compared with the juvenile hypothalamus, *let-7b*, and *let-7g* expression was significantly upregulated in estrous goats (*p* < 0.01). The expression of *let-7b* in the ME and DE hypothalamus and *let-7g* in the DE hypothalamus was downregulated compared with that in juvenile goats (*p* < 0.01). Among the four phases of the first estrous cycle at puberty, the expression of *let-7b* and *let-7g* increased from the PE to the E phase and then decreased. The expression patterns of *let-7b* and *let-7g* in the pituitary (Figure 2d,f) showed greater differences than those in the hypothalamus. Compared with juvenile goats, *let-7b* expression was upregulated in the E and DE pituitaries (*p* < 0.01). In the first estrus cycle, *let-7b* expression increased in response to PE, and reached its highest level in the estrus phase, and then decreased. However, during the diestrus phase, the expression of *let-7b* was upregulated again. The expression of *Let-7g* was upregulated in the PE and E pituitary tissues than those in the JUV pituitary. Like *let-7b*, the expression of *let-7g* in the estrus phase was highest during the first estrus cycle.

### 3.2. Lin28b Affects the Expression of GnRH1, Kiss1 and Gpr54

**Figure 3:**
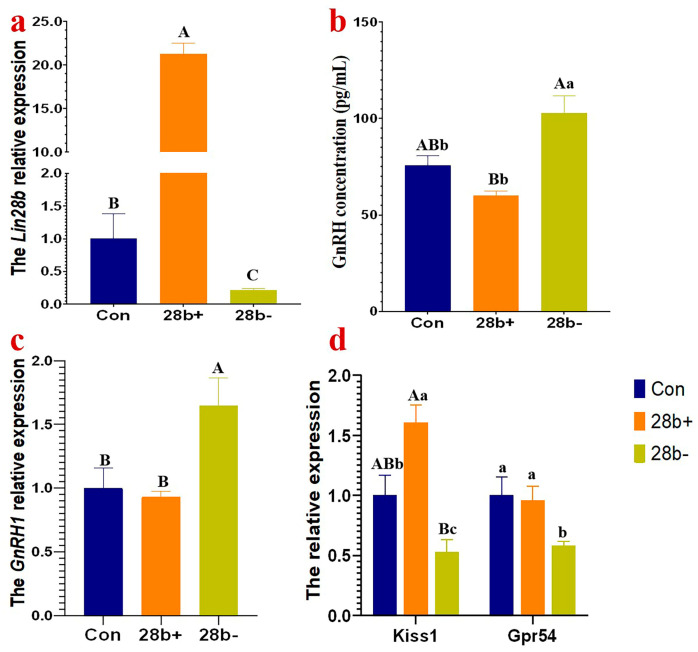
The *GnRH1*, *Kiss1*, and *Gpr54* expression and GnRH concentration in Lin28b+ and Lin28b− GT1-7 cells. (**a**), The *Lin28b* mRNA relative expression. (**b**), The GnRH concentration in the supernatant. (**c**), The *GnRH1* mRNA relative expression. (**d**), The relative expression of *Kiss1* and *Gpr54*. The different capital letters above the columns chart mean a significant difference with *p* < 0.01, and the different small letter means a significant difference with *p* < 0.05 in multiple comparisons.

We investigated whether *Lin28b* inhibits GnRH production in the GnRH-expressing GT1-7 cells by altering *Lin28b* expression. The level of *Lin28b* mRNA abundance was investigated in the GT1-7 cells after transfected with pEGFP-N3-Lin28b, the pSilencer-Lin28b, the empty vectors, and the pSilencer-mimic vector (Supplementary Figure S2). The *Lin28b* mRNA abundance increased approximately 20-fold after pEGFP-N3-*Lin28b* (*p* < 0.01), and that of pSilencer-*Lin28b* decreased to 20% after transfection (*p* < 0.01) (Figure 3a). The GnRH concentration and the *GnRH1* mRNA abundance in Lin28b+ cells were lower than those in control cells without significant difference (*p* = 0.114, *p* = 0.634, respectively). But in Lin28b− cells, the GnRH concentration and the *GnRH1* mRNA level increased significantly in Lin28b− cells (*p* < 0.05, *p* < 0.01, respectively) (Figure 3b,c). *Kiss1* expression was significantly upregulated in Lin28b+ cells and downregulated in Lin28b− cells compared with control cells (*p* < 0.05) (Figure 3d). However, the *Gpr54* expression was only significantly downregulated in Lin28b− cells (*p* < 0.05). In brief, *Lin28b* inhibits *GnRH1* expression, but *GnRH1* expression decreases only slightly in Lin28b+ cells, which indicates a complex regulation. Out of expectation, *Lin28b* enhances Kiss1/Gpr54 signaling, which promotes GnRH expression according to previous studies [9,10,11].

### 3.3. Lin28b Affects the Expression of Genes Related to Glycolysis

**Figure 4:**
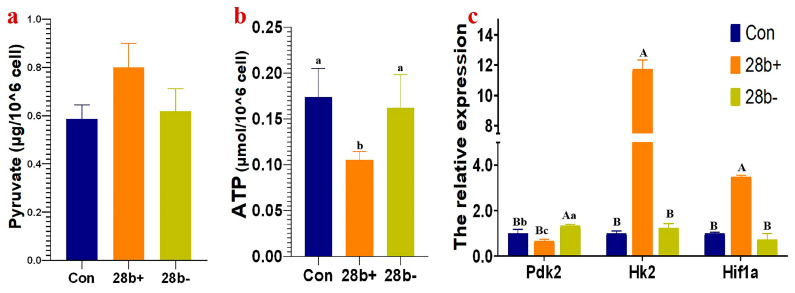
The pyruvate and ATP content and mRNA expression of related genes in Lin28b+ and Lin28b− GT1-7 cells. (**a**), The pyruvate content. (**b**), The ATP content. (**c**), The relative expression of *Pdk2*, *Hk2* and *Hif1a*. The different capital letters above the columns chart mean a significant difference with *p* < 0.01, and the different small letter means a significant difference with *p* < 0.05 in multiple comparisons.

*Lin28b* inhibits *GnRH1* expression and regulates glucose metabolism [21,22,23,24]. We detected the pyruvate and ATP contents (Figure 4a,b) and the mRNA abundance of three genes, *Hk2*, *Hif1a*, and *Pdk2*, related to glycolysis (Figure 4c) in Lin28b+ and Lin28b− GT1-7 cells. The pyruvate content increased (*p* = 0.07), and ATP content decreased (*p* < 0.05) in Lin28b+ cells, but the contents of pyruvate and ATP did not change in Lin28b− cells (Figure 4a,b). The expression of *Hk2*, encoding hexokinase-2, and *Hif1a*, encoding hypoxia-inducible factor 1α, was upregulated in Lin28b+ cells (*p* < 0.01) but did not change in Lin28b− cells. The mRNA expression of *Pdk2*, encoding pyruvate dehydrogenase kinase isozyme 2 (PDK2), was significantly downregulated and upregulated in Lin28b+ and Lin28b− cells, respectively (*p* < 0.05). Therefore, in summary, *Lin28b* overexpression promotes glycolysis but leads to decreased ATP content. Glycolysis is not affected by the downregulated expression of *Lin28b*.

### 3.4. The miRNA Expression Pattern Was Affected by Lin28b

**Figure 5:**
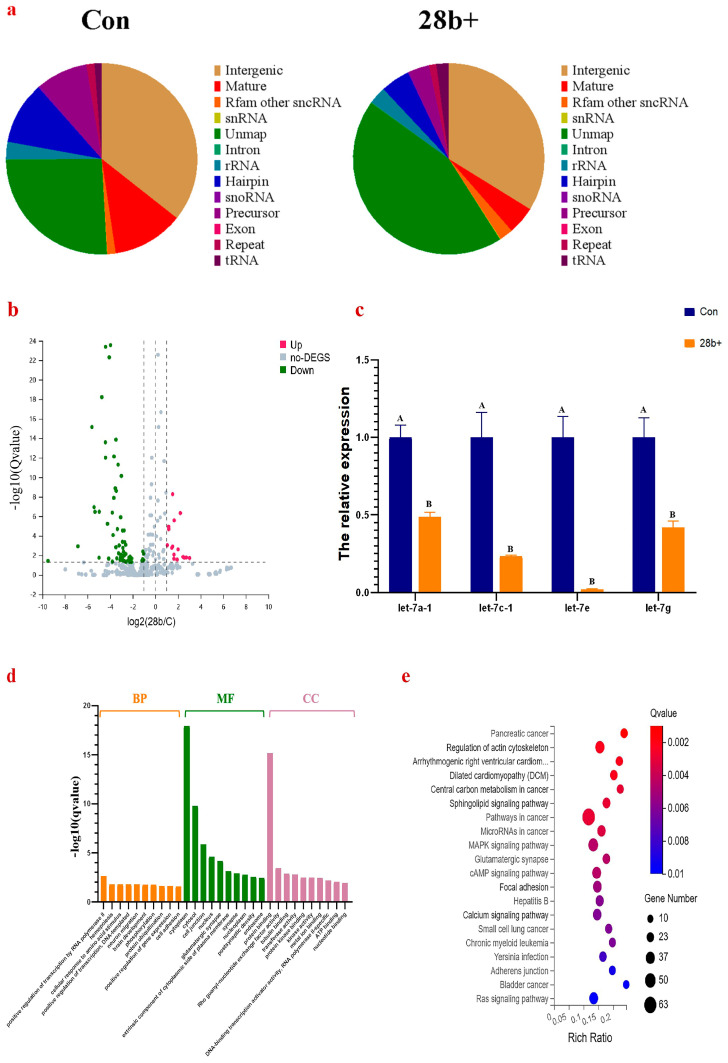
*Lin28b* overexpression in GT1-7 cells affected the miRNA expression profile. (**a**), The classification composition of small RNA in GT1-7 cells. (**b**), Volcanic map of the DEMIs in GT1-7 cells. (**c**), Verification of DEMIs. The different capital letters above the columns chart mean significant difference with *p* < 0.01. (**d**,**e**), GO and KEGG enrichment analysis of the target genes of DEMIs.

*Lin28b* regulates the maturation of the *let-7* miRNA family. To discover additional information about GnRH expression and regulation, the miRNA expression profiles of Lin28b+ GT1-7 cells and control cells were analyzed using small RNA sequencing. After quality control screening, on average, 32.57 million and 30.07 million clean reads were obtained from the small RNA libraries of Lin28b+ and control cells, respectively. A total of 61.29% and 85.14% of the clean reads from the Lin28b+ and control cell libraries were mapped to the mouse genome, respectively. Based on the small RNA classification, *Lin28b* overexpression changed the classification composition of small RNAs in GT1-7 cells (Figure 5a). The percentage of unmapped small RNAs was significantly greater in Lin28b+ cells (*Lin28b+* vs. Con, 43.94% vs. 25.69%), and the percentage of mature miRNAs was significantly lower in Lin28b+ cells (*Lin28b+* vs. Con, 4.74% vs. 12.02%). These changes may be related to the fact that *Lin28b* disturbs the process of miRNA maturation.

A total of 426 miRNAs, containing 98 novel miRNAs, were found, and 77 differentially expressed miRNAs (DEMIs) (|Log_2_FoldChange| > 1, *Q* value < 0.05) were selected, among which 16 were upregulated, and 61 were downregulated (Figure 5b). All the identified DEMIs are shown in Supplementary Table S2. Fourteen members of the *let-7* miRNA family were found to be expressed in GT1-7 cells. The expressions of *let-7e*, *let-7d*, *let-7c-1*, *let-7i*, *let-7a-1*, and *let-7f-1* were significantly downregulated, and the expression of four members was tested using qPCR to verify the data from transcriptome analysis data (Figure 5c).

The target genes of the 77 DEMIs were enriched with GO and KEGG enrichment terms (Figure 5d,e). The top GO terms in the biological process, cellular component, and molecular function categories were positive regulation of transcription by RNA polymerase II, cytoplasm, and protein binding, respectively (Figure 5d). The GO terms also included glutamatergic synapses, positive regulation of gene expression, transmembrane receptor protein tyrosine kinase signaling pathway, protein kinase binding, ATP binding, etc. (Supplementary Table S3). The KEGG pathway analysis was also conducted to further investigate the role of the target genes of the significant DEMIs (Figure 5e). The significantly enriched KEGG pathway categories of the target genes of DEMIs were presented in Supplementary Table S3 (*Q* value ≤ 0.05). Some signaling pathways, for instance, central carbon metabolism in cancer, microRNAs in cancer, the MAPK signaling pathway, the PI3K-Akt signaling pathway, and the mRNA surveillance pathway, related to the known function of *Lin28b* (tumorigenesis, glucose metabolism, and regulation of mRNA expression) were significantly enriched. Similarly, some signaling pathways involved in the GnRH expression, such as the glutamatergic synapse, calcium signaling pathway, sphingolipid signaling pathway [36], HIF-1 signaling pathway [37], arginine biosynthesis pathways [38], were also enriched.

**Figure 6:**
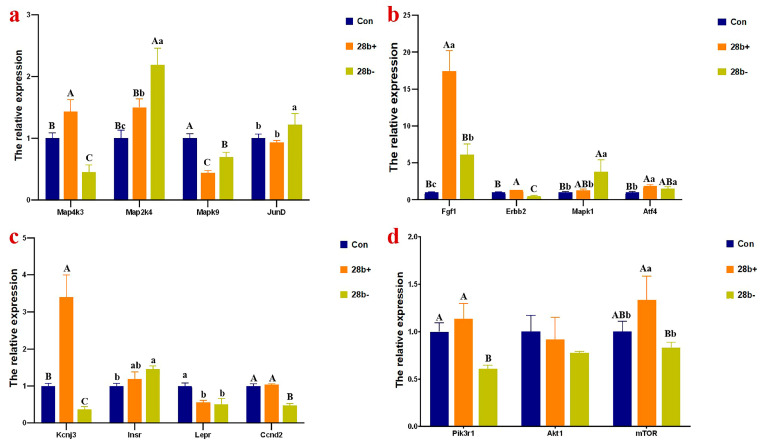
The mRNA expression of several target genes of DEMIs and several key genes in the signaling pathway. (**a**), *Map4k3*, *Map2k4*, *Mapk9*, and *JunD*. (**b**), *Fgf1*, *Erbb2*, *Mapk1*, and *Atf4*. (**c**), *Kcnj3*, *InsR*, *Lepr*, and *Ccnd2*. (**d**), *Pik3r1*, *Akt1*, and *mTOR*. The different capital letters above the columns chart mean a significant difference with *p* < 0.01, and the different small letter means a significant difference with *p* < 0.05 in multiple comparisons.

Several target genes of DEMIs and several key genes involved in the MAPK signaling and the PI3K-mTOR signaling pathway were selected to detect the mRNA expression levels via qPCR (Figure 6). The expression of *Map4k3*, *Erbb2*, and *Kcnj3* were significantly upregulated in Lin28b+ cells (*p* < 0.01) and downregulated in Lin28b− cells (*p* < 0.01), respectively. The mRNA abundance of *Map2k4*, *Fgf1*, and *Atf4* significantly raised whether *Lin28b* was upregulated or downregulated. The expression of *Mapk9* and *Lepr* was downregulated in Lin28b+ and Lin28b+ cells. The expression of *JunD* and *Mapk1* was significantly upregulated in Lin28b− cells, and that of *Ccnd2* and *Pik3r1* was significantly downregulated in Lin28b− cells, respectively. The transcript levels of all four genes were not affected by *Lin28b* overexpression. The *mTOR* gene expression level increased significantly in Lin28b+ cells but did not decrease in Lin28b− cells. *Map4k3*, *Map2k4*, *Mapk9*, *Kcnj3*, *Ccnd2* and *Pik3r1* are the target genes of *let-7* miRNAs. Therefore, their mRNA expression is regulated at least in part by *Lin28b. Fgf1*, *Erbb2*, *Mapk1*, *Atf4*, *Kcnj3*, *Lepr*, *Ccnd2*, *Akt1*, and *mTOR* are the target genes of other DEMIs. These fifteen genes are the key genes in the MAPK signaling pathway or the PI3K-mTOR signaling pathway and participate in glucose metabolism.

### 3.5. GnRH1 mRNA Expression Was Inhibited by Blocking the mTOR Signaling

**Figure 7:**
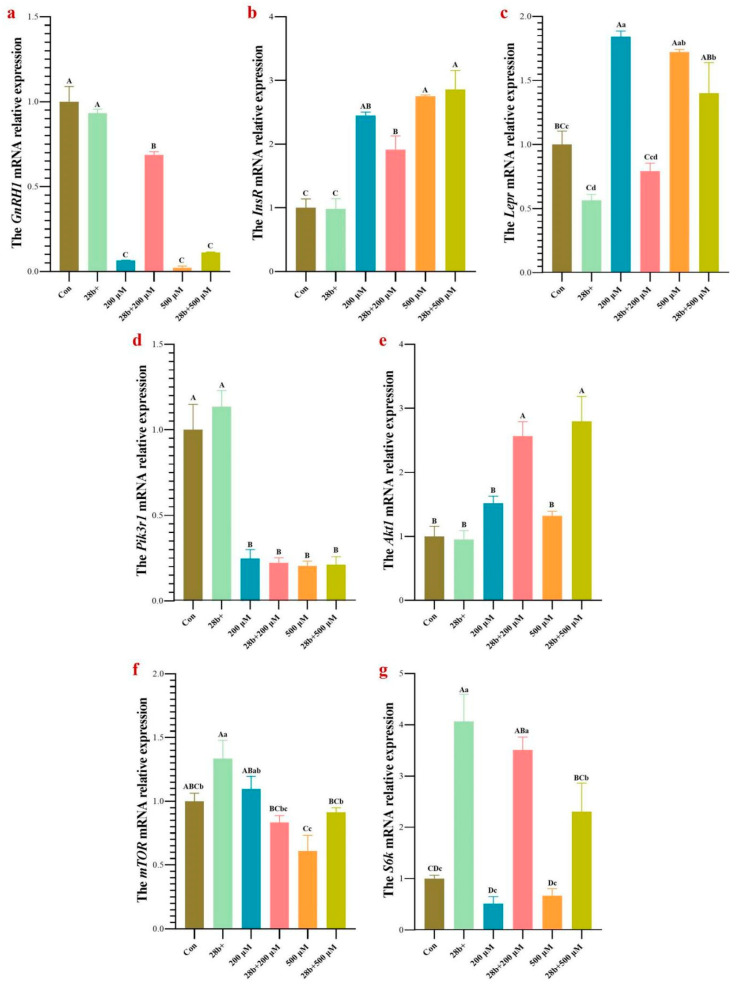
The mRNA relative expression of *GnRH1* and genes related to the mTOR signaling pathway in GT1-7 cells with rapamycin treatment and/or *Lin28b* overexpression. (**a**), *GnRH1*. (**b**,**c**), *InsR* and *Lepr*. (**d**,**e**), *Pik3r1* and *Akt1*. (**f**,**g**), *mTOR* and *S6k*. The different capital letters above the columns chart mean a significant difference with *p* < 0.01, and the different small letter means a significant difference with *p* < 0.05 in multiple comparisons. Con, control GT1-7 cells; 28b+, the GT1-7 cells with *Lin28b* overexpression; 200 μM and 500 μM, the GT1-7 cells treated with 200 μM rapamycin or 500 μM rapamycin, respectively; 28b + 200 μM, the GT 1-7 cells treated with 200 μM rapamycin and *Lin28b* overexpression at the same time; 28b + 500 μM, the GT 1-7 cells treated with 500 μM rapamycin and *Lin28b* overexpression at the same time.

The above results demonstrated that the mRNA expression of several genes related to the PI3K-mTOR signal pathway changed in GT1-7 cells as *Lin28b* expression was altered. We used rapamycin to block the mTOR signaling and/or upregulated *Lin28b* in GT1-7 cells and detected the mRNA expression of *GnRH1*. As shown in Figure 7a, the *GnRH1* mRNA abundance was significantly decreased in the GT1-7 cells treated with 200 μM or 500 μM rapamycin, respectively (*p* < 0.01), and the higher the concentration of rapamycin was, the lower the expression level of *GnRH1* was. Surprisingly, *Lin28b* overexpression during rapamycin treatment increased the expression level of *GnRH1*, especially in the GT1-7 cells treated with 200 μM rapamycin (*p* < 0.01). When GT1-7 cells were treated with 500 μM rapamycin, *Lin28b* overexpression did not significantly rescue *GnRH1* expression.

### 3.6. Rapamycin Altered the Expression of Genes Related to the mTOR Signaling Pathway

Several genes related to mTOR signaling, *Lepr*, *InsR*, *mTOR*, *S6k*, *Pik3r1*, and *Akt1*, were selected for quantification of mRNA expression levels via qPCR (Figure 7b–g). The mRNA expression of *Lepr* and *InsR* was upregulated in the GT1-7 cells treated with rapamycin. *Lin28b* overexpression inhibited *Lepr* expression and could weaken the promoting effect of rapamycin on *Lepr* expression, especially in the GT1-7 cells treated with 200 μM rapamycin. However, the expression of *InsR* was not affected by *Lin28b* overexpression, regardless of the presence of rapamycin. Similarly, the *Pik3r1* mRNA abundance decreased in GT1-7 cells treated with rapamycin (*p* < 0.01) and was not affected by *Lin28b* overexpression (Figure 7d). *Lin28b* overexpression alone did not affect the expression level of *Akt1*, and rapamycin treatment slightly upregulated the *Akt1* expression level. However, *Lin28b* overexpression and ramamycin treatment simultaneously upregulated *Akt1* expression (*p* < 0.01) (Figure 7e). The *mTOR* expression was significantly downregulated in GT1-7 cells treated with 500 μM rapamycin. And *Lin28b* overexpression could weaken the inhibitory effect of rapamycin on *mTOR* expression in Lin28b+ 500R GT1-7 cells. Contrary to our expectations, the expression of *S6k*, a gene just downstream of mTORC1 and upstream of mTORC2, was reduced slightly when the GT1-7 cells treated with rapamycin. *Lin28b* overexpression during rapamycin treatment significantly increased the *S6k* mRNA abundance (*p* < 0.01) (Figure 7g).

In brief, rapamycin upregulated the mRNA expression of *Lepr*, *InsR*, and *Akt1* (slightly) and downregulated the expression of *Pik3r1*, *S6k*, and *mTOR* (500 μM rapamycin). When mTOR signaling was blocked by rapamycin, *Lin28b* overexpression upregulated the expression of *Akt1*, *S6k*, and *mTOR* (500 μM rapamycin), downregulated *Lepr* expression, and did not affect the expression of *InsR* and *Pik3r1*. Therefore, *Lin28b* may rescue *GnRH1* expression in rapamycin-treated GT1-7 cells by activating *Akt1* and its downstream genes in the PI3K-mTOR signaling pathway.

## 4. Discussion

In the present study, *Lin28b* and *let-7b/g* miRNA expression in the hypothalami of juvenile and pubertal goats were detected. The possible signaling pathways underlying the regulatory effect of *Lin28b* on GnRH expression in the GnRH-producing cell model GT1-7 were investigated.

*Lin28b* was expressed in the hypothalami of goats, which was consistent with what has been observed in mice [14], rats, and monkeys [8]. However, the expression pattern in the pituitary was not consistent. *Lin28b* was expressed in the mouse pituitary gland but not in the rat or goat pituitary gland. *GnRH1* expression significantly increased in the goat pubertal hypothalamus, consistent with the previous reports that GnRH pulse release rapidly increased when goats reached puberty. In contrast, the expression level of *Lin28b* in the pubertal hypothalami decreased during pubertal development, consistent with what has been observed in rats and monkeys [8]. Therefore, the decreased expression of *Lin28b* in the pubertal hypothalamus indicates a role in the inhibition of GnRH production. The *GnRH1* mRNA abundance and GnRH concentration significantly increased in GT1-7 cells when *Lin28b* was downregulated. However, when *Lin28b* was upregulated, the expression of *GnRH1* did not decrease as predicted, and the GnRH concentration was decreased slightly (*p* = 0.055), which was different from the results obtained for *Lin28a* overexpression in GT1-7 cells. *Lin28a* overexpression in GT1-7 cells significantly reduced the GnRH concentration, and disruption of *Lin28a* expression increased GnRH production [11]. These unexpected results may be attributed to the activation of signaling pathways that increase the expression of *GnRH1* to rescue or balance the inhibitory effect of *Lin28b* on stabilizing GnRH secretion. Even with high homology, *Lin28a* and *Lin28b* have different functions and regulatory patterns.

*Lin28b* overexpression stimulated the expression of *Kiss1* but had no impact on *Gpr54* mRNA expression. However, *Kiss1* and *Gpr54* were all downregulated when *Lin28b* was interfered with. Therefore, *Lin28b* is positively correlated with *Kiss1*/*Gpr54* signaling, and which is different from the results in GT1-7 cells with *Lin28a* overexpression [11]. However, kisspeptins can promote GnRH secretion by binding to Gpr54 through activating several signaling pathways [10,39]. Therefore, it seems that *Kiss1*/*Gpr54* signaling is not the dominant pathway in the regulatory effect of *Lin28b* on GnRH secretion. The promoting effect of *Kiss1*/*Gpr54* signaling on GnRH induced by *Lin28b* is blocked or weakened by other signaling pathways regulated by *Lin28b*.

The pyruvate content increased, and the ATP content decreased in Lin28b+ GT1-7 cells. Surprisingly, the pyruvate and ATP contents were not affected by *Lin28b* knockdown. Concurrently, the mRNA abundances of genes related to glycolysis, such as *Hif1a* and *Hk2*, were significantly changed. These observations support the concept that *Lin28b* promotes glycolysis in GT1-7 cells. And *Lin28a* has similar functions [40,41]. The hexokinase-2, *Hk2* gene product, mediates the initial step of glycolysis by catalyzing the phosphorylation of D-glucose to D-glucose 6-phosphate and is a key rate-limiting enzyme in glycolysis. HIF-1α can bind to the promoter of *Hk2* and promote its transcription [42]. Therefore, *Lin28b* is predicted to upregulate *Hif1a* expression, which subsequently promotes *Hk2* mRNA expression. Moreover, *Hk2* is the target gene of *miR-24-2-5p*, a downregulated DEMI in Lin28b+ cells (Supplement Table S2). Therefore, *Lin28b* may also upregulate *Hk2* expression by inhibiting the maturation of *miR-24-2-5p*. Therefore, *Lin28b* might promote *Hk2* expression to increase hexokinase production, which promotes more glucose entry into the glycolytic pathway and increases pyruvate production. In *Lin28a*-overexpressed HEK293 cells, *Hk2* expression was also upregulated, but *Hif1a* expression was not significantly different [41].

**Figure 8:**
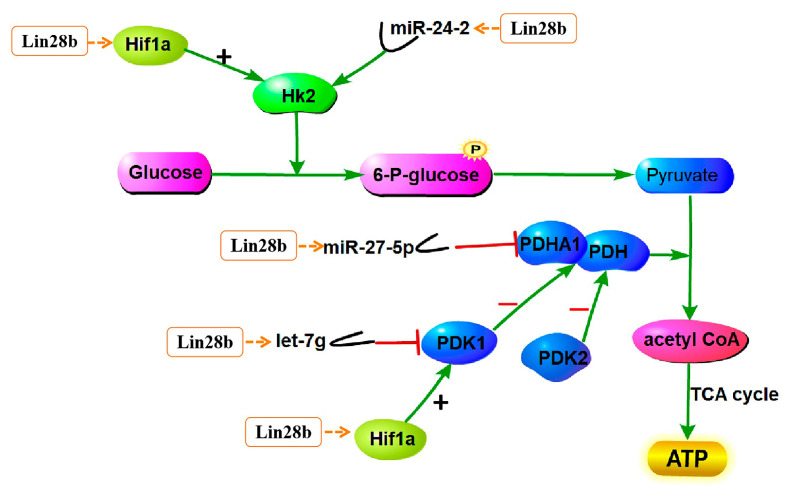
The predicted regulatory diagram of *Lin28b* and glycolysis.

Pyruvate dehydrogenase kinase isozyme 2 (PDK2) phosphorylates pyruvate dehydrogenase (PDH) to inhibit the activity of the pyruvate dehydrogenase complex, which reduces mitochondrial pyruvate metabolism [43]. In Lin28b+ GT1-7 cells, the *Pdk2* mRNA expression was significantly downregulated. The *Pdha1* gene, encoding a subunit of the PDH, is the target gene of *miR-27b-5p*, a downregulated DEMI in Lin28b+ cells (Supplement Table S2). Through the dual regulation of *Pdk2* and *miR-27b-5p*, PDH activity might be promoted. In addition, in *Lin28a*-overexpressed HEK293 cells, PDH expression was significantly increased [41]. Based on these findings, *Lin28b* overexpression promotes more pyruvate entry into the TCA cycle and ATP production. On the contrary, in fact, pyruvate content was increased, and ATP content decreased in Lin28b+ cells. So, there are other genes or pathways to regulate PDH. It was reported that Hif1a, via its direct target pyruvate dehydrogenase kinase 1 (PDK1), negatively regulates PDH to shut down pyruvate entering into the TCA cycle under hypoxic conditions [44,45,46]. In some tumor cells, *Lin28a* and *Lin28b* regulated *Pdk1* through *let-7g* to promote glycolysis and inhibit PDH activity [40]. *Let-7g* was significantly downregulated, and *Hif1a* was increased in Lin28b+ cells. In GT1-7 cells, if *Lin28b* negatively regulates PDH via *Hif1a* and *let-7g*, the pyruvate entering into the TCA cycle is inhibited or reduced, leading to decreased ATP content and the accumulation of pyruvate. So, in Lin28b+ cells, it is dominant that *Lin28b* modulates PDH through *Hif1a* and *let-7g*. So, it would be a complex or dual regulation of *Lin28b* on glycolysis and TCA cycle (Figure 8). Inconsistent with our expectation, the pyruvate and ATP contents, the *Hk2* and *Hif1a* transcript levels were not affected by *Lin28b* knockdown. To summarize, *Lin28b* overexpression promoted glycolysis, which was not affected by *Lin28b* downregulation. These results indicate that *Lin28b* is not the main limiting factor but rather a stimulus for glycolysis.

The MAPK and PI3K-Akt signaling pathways were significantly enriched by the target genes of significant DEMIs. The MAPK and PI3K signaling participated in the regulation of GnRH synthesis by 5-hydroxytryptamine (5-HT), insulin, docosahexaenoic acid, and palmitate [47,48,49]. The inhibition of p38MAPK caused an increase in the peripheral concentration of GnRH3 in the lenok [50]. The MAPK pathway played a role in the blocking effect of *Lin28a* on miRNA maturation by enhancing the phosphorylation and binding of the Dicer cofactor, TAR RNA-binding protein (TRBP), and Dicer, to Lin28a to establish interactions and stabilize the interaction of Lin28a/TRBP/Dicer protein complex [51]. *Map4k3*, *Map2k4*, and *Mapk9* are the target genes of *let-7* miRNA, and *Mapk9* is also a target gene of *novel-miR-205*, an upregulated DEMI in Lin28b+ cells. Therefore, *Lin28b* may regulate the expression of members of MAPK signaling components via *let-7* miRNA or other miRNAs and thereby inhibit GnRH production. The PI3K/Akt signaling involves GnRH secretion in several species. Ye et al. identified the differentially abundant proteins (DAPs) in the hypothalamus between pubertal and prepubertal goats, and several DAPs were enriched in the PI3K/Akt/mTOR pathway [52]. Activation of the PI3K/Akt pathway protected GnRH neurons from hypoxia-reoxygenation (HR)-induced decrease in GnRH level through forkhead box protein O3a (FoxO3a) [53]. In *Gobiocypris rarus*, 17α-methyltestosterone disturbed the GnRH and FSH/LH concentrations via the PI3K/Akt/FoxO3a signaling pathway [54]. In GT1-7 cells, 5-hydroxytryptamine receptor 1A (HTR1A) blocked PI3K/Akt and MAPK/ERK pathways to silence the transcription of chromobox 4 (CBX4) and led to the degradation of the polycomb-repressive complex 1 (PRC1), weakening histone H2A lysine-119 ubiquitinations at the promoter of *GnRH1*, and ultimately enhancing *GnRH1* transcription [49]. Cbx2, a member of PRC1, was the target gene of miR-24-1-5p, a downregulated DEMI in Lin28b+ cells. Additionally, HTR7, another receptor for 5-HT, was the target gene of miR-24-2, which also was a downregulated DEMI in Lin28b+ cells. So, it is predicted that *Lin28b* regulates *GnRH1* expression by regulating the expression of genes related to the MAPK and PI3K/Akt signaling pathway. However, further functional studies on the expression regulation of *Lin28b* on *Pik3r1*, *map2k4*, and *Cbx2* are necessary.

Blocking the mTOR signaling by 200 μM or 500 μM rapamycin in GT1-7 cells inhibited *GnRH1* expression, and the higher the concentration of rapamycin was, the lower the mRNA abundance of *GnRH1* was. *Lin28b* overexpression increased the *GnRH1* transcript level in the 200 μM rapamycin-treated GT1-7 cells but not in the 500 μM rapamycin-treated cells. It suggests that *Lin28b* rescues the expression inhibition of *GnRH1* induced by lower concentrations of rapamycin. The mRNA expression of several genes, *Lepr*, *InsR*, *mTOR*, *S6k*, *Pik3r1*, and *Akt1*, participating in the PI3K-mTOR pathway was examined via RT-qPCR. Blocking mTOR signaling promoted the expression of *InsR* and *Lepr*. *Lin28b* overexpression inhibited *Lepr* expression and weakened the promotive effect of rapamycin on *Lepr* but did not affect the *InsR* transcript level. Rapamycin treatment inhibited *Pik3r1* expression, which was not affected by *Lin28b*. *Lin28b* overexpression promoted the expression of *S6k* and weakened the inhibitory effect of rapamycin on *S6k*. A higher concentration of rapamycin inhibited *mTOR* mRNA expression in GT1-7 cells, which could be rescued by *Lin28b* overexpression. Therefore, *Lin28b* should have a positive regulatory effect on *S6k* expression. The mRNA expression of the *Atk1* gene tended to increase in rapamycin-treated cells but increased significantly in Lin28b+R cells, which suggested that *Lin28b* might activate *Akt1* when mTOR signaling was blocked. In brief, *Lin28b* promotes the expression of *GnRH1* through *mTOR* and *S6k*, and when the mTOR signaling is blocked, *Lin28b* promotes *Akt1* and *S6k* to regulate GnRH expression.

**Figure 9:**
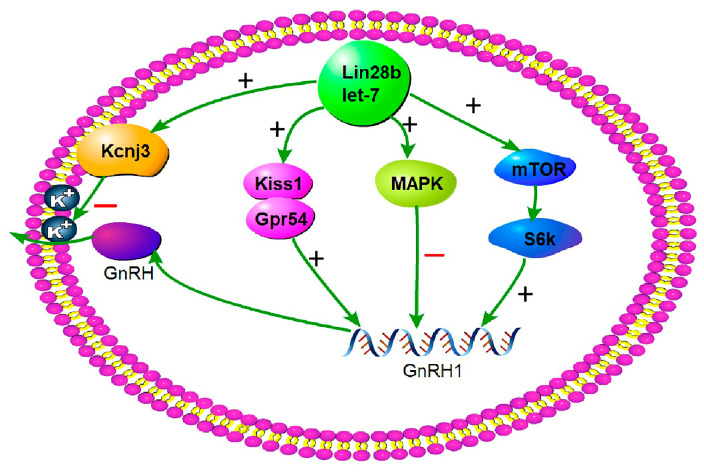
The overview of *Lin28b* and the signaling pathways that are involved in GnRH expression.

Unfortunately, our results lack protein expression data for key genes in the abovementioned signaling pathways. Despite its limitation, this study suggests that *Lin28b-let-7* positively regulates the mTOR and Kiss1/Gpr54 signaling to upregulate GnRH expression and positively regulate the MAPK signaling to downregulate GnRH expression (Figure 9).

## 5. Conclusions

*Lin28b* mRNA abundance in the hypothalamus decreases during goat pubertal development. *Lin28b* inhibits *GnRH1* mRNA expression in the GnRH model neurons, GT1-7 cells. In vitro, *Lin28b* promotes glycolysis and stimulates the Kiss1/Gpr54 signaling, the MAPK, and AKT-mTOR signaling pathways, which are all related to glucose metabolism, and at least partially through *let-7* miRNAs. Therefore, *Lin28b* modulates GnRH production by regulating glucose metabolism through a complex network.

## Data Availability

The data presented in this study are available upon request from the corresponding author.

## Supplementary Materials

The following supporting information can be downloaded at https://www.mdpi.com/article/10.3390/ani15020120/s1. Figure S1: The schematic diagram; Figure S2: The *Lin28b* mRNA expression in GT1-7 cells with *Lin28b* up- and down-regulated expression; Table S1: Primer sequences; Table S2: Differentially expressed miRNA; Table S3: GO and KEGG analysis of DEMIs.
