# Supplementary material for: Lin28b-let-7 Modulates mRNA Expression of GnRH1 Through Multiple Signaling Pathways Related to Glycolysis in GT1-7 Cells
Source: Animals (Basel). 2025 Jan 7;15(2):120. doi: 10.3390/ani15020120 (PMC11758636; doi:10.3390/ani15020120)
Supplement: Supplementary file 1 [file animals-15-00120-s001.zip › animals-3355675-supplementary/Supplementary Figures.pdf]

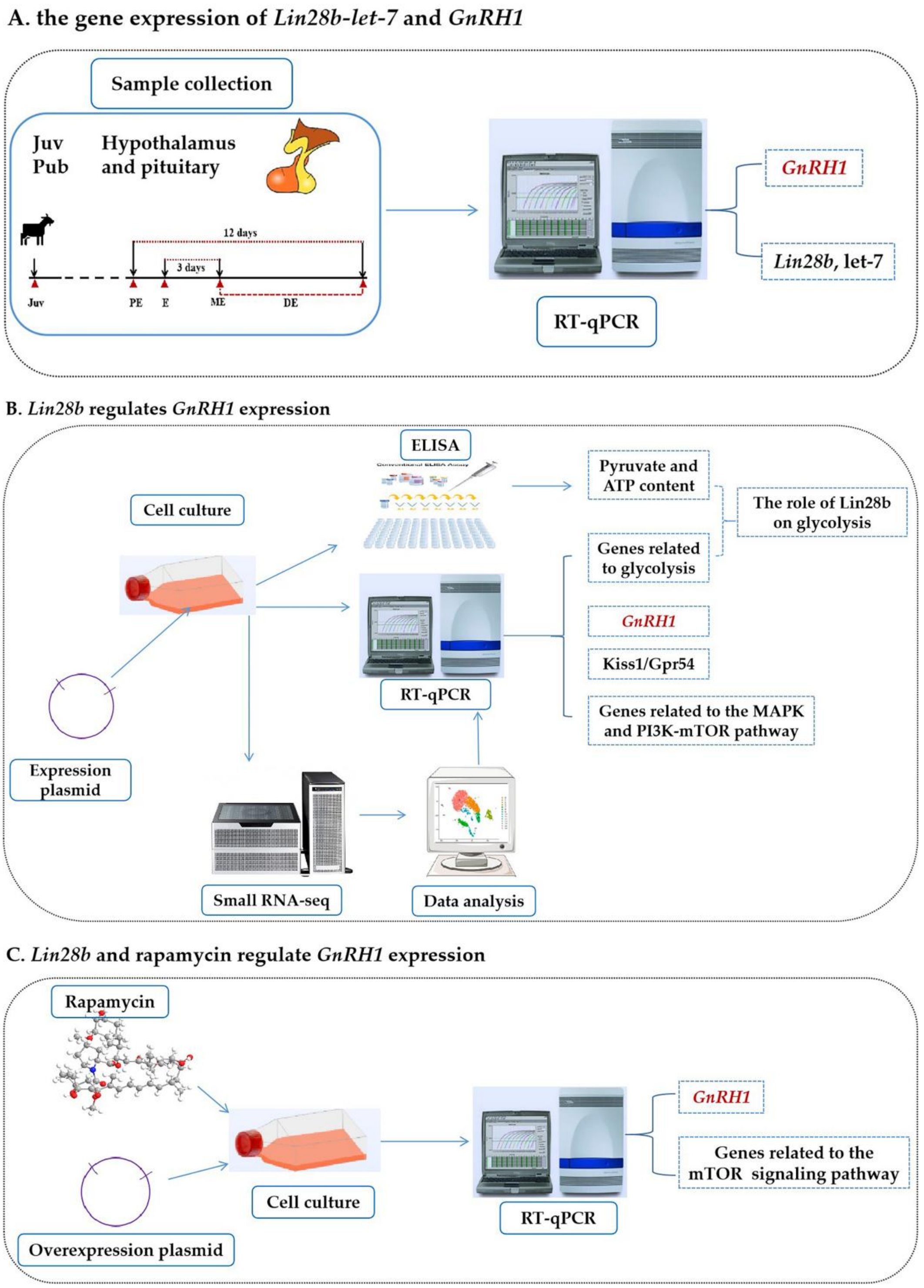

Supplementary Figure 1. The schematic diagram. A, gene expression detection of *Lin28b*, *let-7* and *GnRH1* in the hypothalamus and pituitary tissues of Juvenile and puberty goats. B, upregulated and downregulated expression of *Lin28b* in GT1-7 cells regulates *GnRH1* in GT1-7 cells. C, *Lin28b* overexpression and rapamycin treatment regulate *GnRH1*in GT1-7 cells.

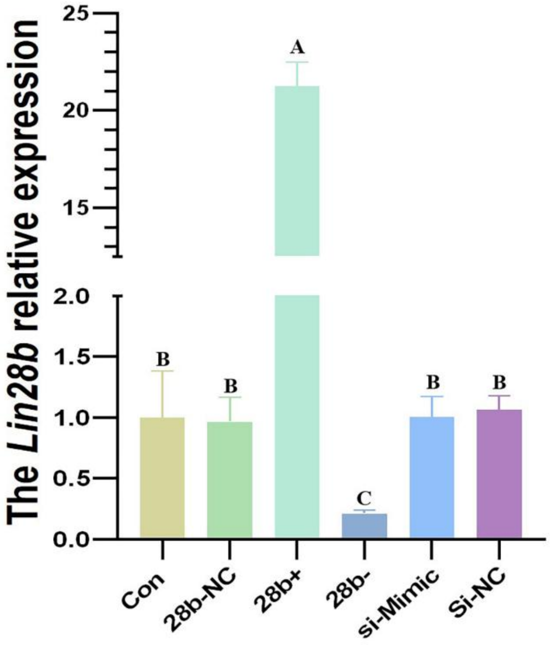

Supplementary Figure 2. The *Lin28b* mRNA expression in GT1-7 cells with *Lin28b* up- and down-regulated expression.
